# Discovery of Novel Biomarkers with Extended Non-Coding RNA Interactor Networks from Genetic and Protein Biomarkers

**DOI:** 10.3390/ijms251810210

**Published:** 2024-09-23

**Authors:** Gregor Jezernik, Damjan Glavač, Pavel Skok, Martina Krušič, Uroš Potočnik, Mario Gorenjak

**Affiliations:** 1 Center for Human Genetics & Pharmacogenomics, Faculty of Medicine, University of Maribor, Taborska ulica 8, 2000 Maribor, Slovenia; damjan.glavac@mf.uni-lj.si (D.G.); martina.krusic1@um.si (M.K.); uros.potocnik@um.si (U.P.); mario.gorenjak@um.si (M.G.); 2 National-Level Institute for Sustainable Environmental Solutions, Jadranska cesta 28, 2000 Maribor, Slovenia; 3 Department of Molecular Genetics, Institute of Pathology, Faculty of Medicine, University of Ljubljana, Korytkova 2, 1000 Ljubljana, Slovenia; 4 Department of Gastroenterology, Internal Medicine Clinic, University Medical Centre Maribor, Ljubljanska ulica 8, 2000 Maribor, Slovenia; pavel.skok@ukc-mb.si; 5 Faculty of Medicine, University of Maribor, Taborska ulica 8, 2000 Maribor, Slovenia; 6 Department for Science and Research, University Medical Centre Maribor, Ljubljanska ulica 8, 2000 Maribor, Slovenia; 7 Faculty of Chemistry and Chemical Engineering, University of Maribor, Smetanova ulica 17, 2000 Maribor, Slovenia

**Keywords:** gene ontology, non-coding RNA, disease pathogenesis

## Abstract

Curated online interaction databases and gene ontology tools have streamlined the analysis of highly complex gene/protein networks. However, understanding of disease pathogenesis has gradually shifted from a protein-based core to complex interactive networks where non-coding RNA (ncRNA) is thought to play an essential role. As current gene ontology is based predominantly on protein-level information, there is a growing need to analyze networks with ncRNA. In this study, we propose a gene ontology workflow integrating ncRNA using the NPInter V5.0 database. To validate the proposed workflow, we analyzed our previously published curated biomarker datasets for hidden disease susceptibility processes and pharmacogenomics. Our results show a novel involvement of melanogenesis in psoriasis response to biological drugs in general. Hyperpigmentation has been previously observed in psoriasis following treatment with currently indicated biological drugs, thus calling attention to melanogenesis research as a response biomarker in psoriasis. Moreover, our proposed workflow highlights the need to critically evaluate computed ncRNA interactions within databases and a demand for gene ontology analysis of large miRNA blocks.

## 1. Introduction

The advent of stringently curated online databases of gene–gene and protein–protein interactions has elevated gene network analysis from mere literature search to an objective and systematic method. Most importantly, the Gene Ontology Consortium has developed an evidence-based hierarchical system of hypernyms and hyponyms to characterize genes or proteins through the biological processes, molecular functions and cellular components that are related to the gene or protein [1]. Gene ontology (GO) tools play a crucial role in analyzing large lists of genes or proteins by facilitating statistical evaluation to identify over- and underrepresented biological processes. This capability is invaluable for swiftly pinpointing key processes, thus expediting the identification of critical molecular pathways. By leveraging such tools, researchers can conduct more targeted downstream reanalysis or propose functional studies, focusing on the molecular pathways most likely to produce significant results. This approach enhances efficiency and accuracy in biological research, ultimately advancing our understanding of complex biological systems. In addition, gene ontology tools enable meta-analysis of existing biomarkers. The current landscape of biomarker studies presents a very heterogeneous picture of predictive biomarkers with low replications rates. Although this is predominantly due to population differences in ethnic background, the phenotypes investigated usually converge to be very similar despite different genetic components. As such, gene ontology tools can be used to collect published biomarkers from different regional studies and form new hypotheses based on enriched terms or interactor genes.

However, the understanding of disease pathogenesis has been gradually shifting away from a purely Mendelian genetics and protein-based paradigm towards one where non-protein processes with their underlying complex genetics play a much greater role. The aim is to integrate these new layers of data as seamlessly as possible with other types of data [2]. Epigenomics (ex. DNA methylation), expression quantitative loci (eQTL) and non-coding genomic variants affecting splicing have already been incorporated into more modern models of disease pathogenesis. In the last decade, the role of non-coding RNA (ncRNA), particularly miRNA and lncRNA, has further changed the protein-based core, leading to hypotheses such as the competing endogenous RNA (ceRNA) hypothesis [3]. Although evidence and opinions on the ceRNA hypothesis [4] differ, there is undeniably a growing need for comprehensive analysis of miRNA and lncRNA function similar to or supporting gene ontology tools. Due to the protein-based nature of GO, current GO methods cannot accommodate this demand. There have been many attempts at functional annotation of miRNA, such as the miRBase database [5] and the miRNA annotations published by the UCL Institute of Cardiovascular Science [6]. To our best knowledge, protocols incorporating lncRNA and other types of non-coding RNA have been few and good practices for taking advantage of gene-ncRNA and ncRNA-ncRNA interaction databases are still in development. Moreover, taking advantage of ncRNA interaction databases using previously published DNA, RNA and protein biomarkers may aid in forming hypotheses and study designs for targeted biomarker studies and also functional studies.

To achieve this objective, we will reanalyze datasets that we previously curated for the identification of hidden disease susceptibility processes and pharmacogenomics response markers to biological drugs. Our focus will be on identifying significant regulatory loops within the mRNA-miRNA-lncRNA axis, supported by experimental evidence. By leveraging these datasets, we aim to establish examples for future analyses of mRNA-miRNA-lncRNA networks. Furthermore, we aim to use this method to discover or replicate miRNA and lncRNA biomarkers in association with disease pathogenesis and drug response. These analyses will be conducted in conjunction with gene ontology tools to provide comprehensive insights into the functional relevance of identified regulatory loops.

## 2. Results

### 2.1. Proposed Filtering Protocol

For mRNA-miRNA-lncRNA axis generation from a curated list of mRNA and/or protein biomarkers (also known as genes of interest), our proposed protocol suggests first filtering and extracting only one type of ncRNA within the NPInter V5.0 dataset. Next, another type of ncRNA is filtered and extracted so all newly selected biomarkers have at least one interaction with a gene of interest and an interacting miRNA. This approach reduces dataset size and prunes biomarkers with only one interactor, retaining only elements desired for later mRNA-miRNA-lncRNA regulatory loops. These principles similarly apply to network generation from a curated list of ncRNA.

### 2.2. Filtered mRNA-miRNA–Protein Network

**Figure 1:**
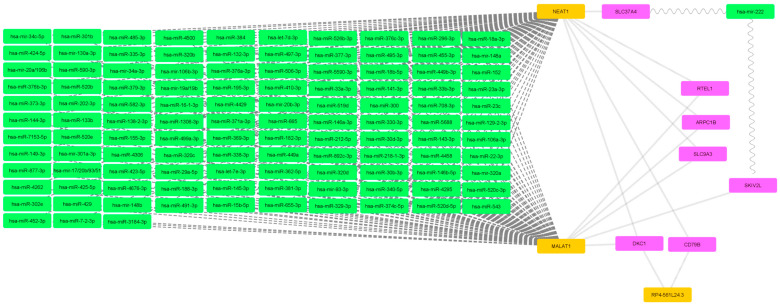
The mRNA-miRNA-lncRNA network of pediatric IBD and IBD-like gene dataset. Magenta nodes represent mRNA, green nodes represent miRNA and orange nodes represent lncRNA. Wavy lines connect mRNA and miRNA, dashed lines connect miRNA and lncRNA while backwards slash lines connect mRNA and lncRNA.

The pediatric IBD and IBD-like genetic marker dataset has produced a mRNA-miRNA–protein network with a significant miRNA block which does not interact with any genes of interest but is regulated by NEAT1 and MALAT1 (Figure 1). Out of 84 genes of interest, only 7 are involved in the resulting network. The filtering threshold for this dataset remains at the default value of 1.

**Figure 2:**
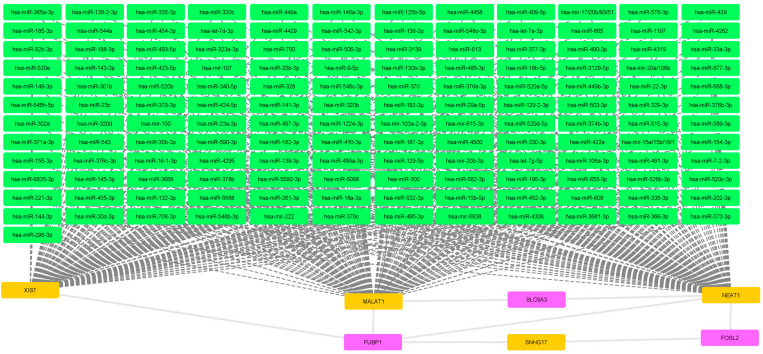
The mRNA-miRNA-lncRNA network of adult complex IBD gene dataset. Magenta nodes represent mRNA, green nodes represent miRNA and orange nodes represent lncRNA. Dashed lines connect miRNA and lncRNA while backwards slash lines connect mRNA and lncRNA.

To produce a processable dataset, stricter filtering with a threshold of 4 was performed for the adult complex IBD dataset (Figure 2). Similar to the pediatric IBD dataset, a major miRNA block that does not interact with any genes of interest has been produced. Interestingly, the miRNA block is governed by NEAT1 and MALAT1 but also XIST. Out of 164 included genes of interest, only 3 were retained following stringent filtering.

**Figure 3:**
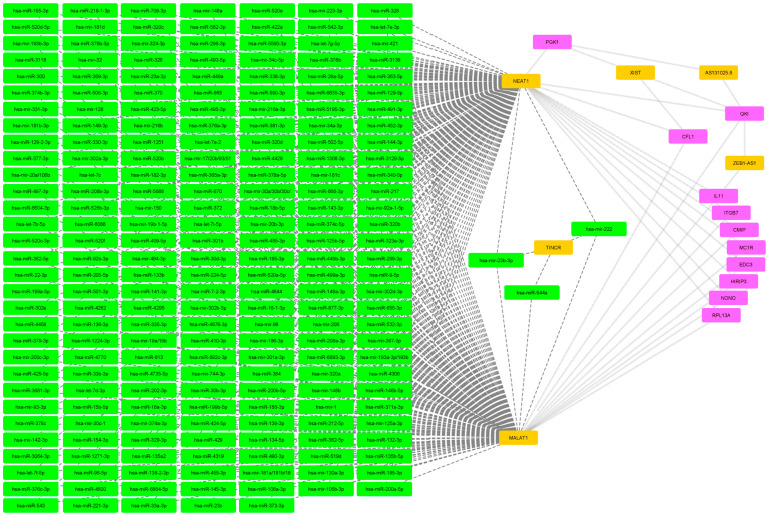
The mRNA-miRNA-lncRNA network of rheumatoid arthritis pharmagenomics dataset. Magenta nodes represent mRNA, green nodes represent miRNA and orange nodes represent lncRNA. Dashed lines connect miRNA and lncRNA while backwards slash lines connect mRNA and lncRNA.

The filtering threshold for the rheumatoid arthritis pharmacogenomics dataset has been set to 4 (Figure 3). Following filtering, 11 of the initial 390 biomarkers of interest were retained. Again, NEAT1 and MALAT1 are governing a large miRNA block.

**Figure 4:**
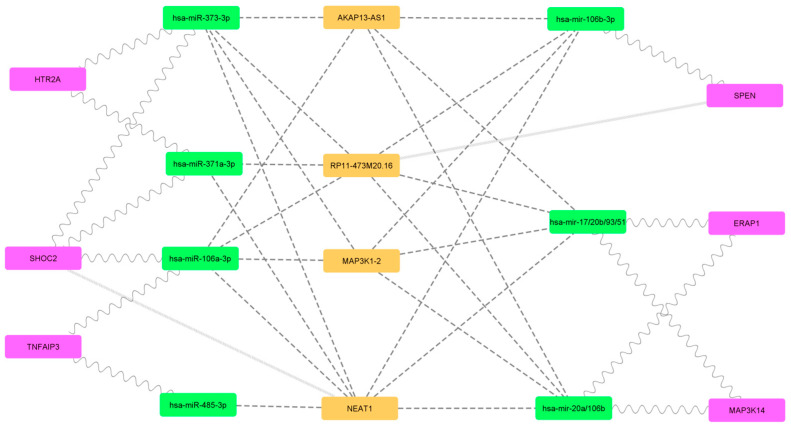
The mRNA-miRNA-lncRNA network of psoriasis pharmagenomics dataset. Magenta nodes represent mRNA, green nodes represent miRNA and orange nodes represent lncRNA. Wavy lines connect mRNA and miRNA, dashed lines connect miRNA and lncRNA while backwards slash lines connect mRNA and lncRNA.

**Figure 5:**
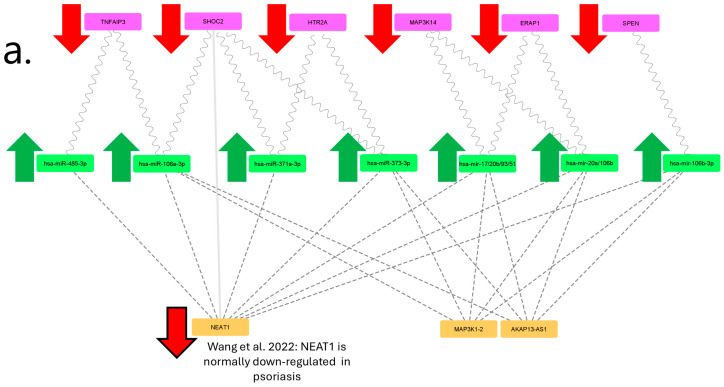

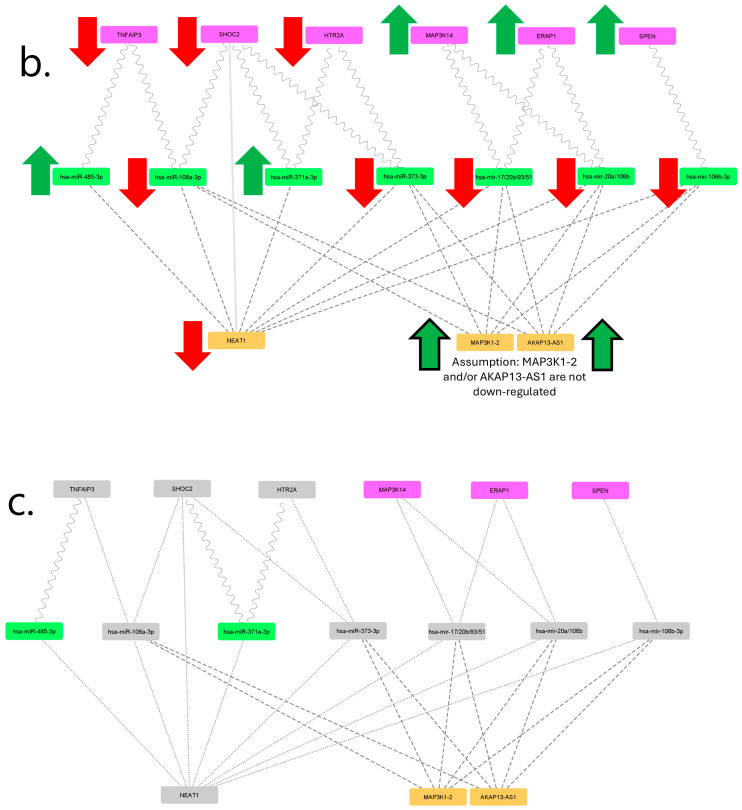
(**a**–**c**) A psoriasis mRNA-miRNA-lncRNA network following gene expression hypothesis. Magenta nodes represent mRNA, green nodes represent miRNA, orange nodes represent lncRNA and gray nodes represent inactive or lowly expressed RNA. Wavy lines connect mRNA and miRNA, dashed lines connect miRNA and lncRNA, backwards slash lines connect mRNA and lncRNA while thin dotted lines represent inactive connections [9].

The filtering threshold for the psoriasis pharmacogenomics dataset remains the default 1 (Figure 4). After pruning of single interactors, 6 of the original 64 genes of interest were retained. The resulting mRNA-miRNA-lncRNA network has only 17 codes and is easily legible with distinguishable levels for each biomarker type. In the existing literature, RP11-473M20.16 has been mentioned in few articles [7,8] and has not yet been associated with psoriasis. Meanwhile, NEAT1 has been shown to be lowly regulated in psoriasis (Figure 5a) [9]. If MAP3K1-2 and AKAP13-AS1 are not down-regulated, this leads to mRNA-miRNA-lncRNA network simplification and a distinct expression profile (Figure 5b). The figure shows that after accounting for known up- and down-regulation expression patterns in psoriasis, only at the endpoint *TNFAIP3*, *SHOC2* and *HTR2A* expression is retained (Figure 5c).

**Figure 6:**
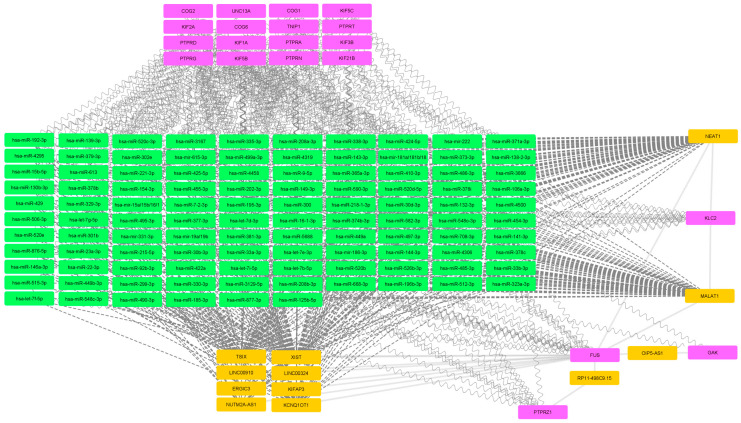
The mRNA-miRNA-lncRNA network of the ALS gene dataset. Magenta nodes represent mRNA, green nodes represent miRNA and orange nodes represent lncRNA. Wavy lines connect mRNA and miRNA, dashed lines connect miRNA and lncRNA while backwards slash lines connect mRNA and lncRNA.

The ALS mRNA dataset was filtered with a threshold set at 3 (Figure 6). Compared to other datasets, 20 out of 38 genes of interests were retained, which is proportionally the highest (52.6%). NEAT1 and MALAT1 are present and produce a major miRNA block. However, in the ALS mRNA dataset, the miRNA block has direct interactions with genes of interest. Moreover, the miRNA block also interacts with eight other lncRNAs (TSIX, XIST, LINC00910, LINC00324, ERGIC3, KIFAP3, NUTM2A-AS1 and KCNQ1OT1) besides NEAT1 and MALAT1.

**Figure 7:**
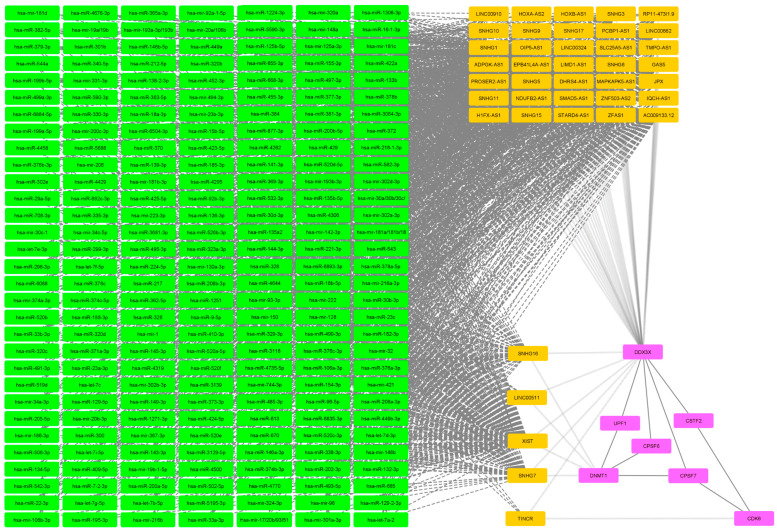
The genes governed by lncRNA interactors of the NEAT1-MALAT1 miRNA block. Magenta nodes represent mRNA, green nodes represent miRNA and orange nodes represent lncRNA. Dashed lines connect miRNA and lncRNA while backwards slash lines connect mRNA and lncRNA.

The large miRNA block that forms whenever the lncRNAs NEAT1 and MALAT1 are present warranted further investigation into whether it represents an underlying commonality between the analyzed disease or pharmacogenomics biomarker or if it is background noise that arises from ncRNA analysis with a sufficient amount of ncRNA input. To this end, we extracted all miRNA names that appear in these miRNA blocks for all presented figures and searched for all mRNAs and proteins that interact with an lncRNA that in turn interacts with at least two miRNA markers from the NEAT1-MALAT1 miRNA blocks. This search would present us with genes affected by lncRNA that are governed by at least two miRNAs from the NEAT1-MALAT1 miRNA block. The pruned network is presented in Figure 7. Analyzed miRNAs and their interactors are summarized as lists in Table 1.

### 2.3. Gene Ontology Analysis

Following network pruning, genetic markers retained after mRNA-miRNA-lncRNA network generation were expanded with their interactors on the gene–gene and protein–protein level using the BioGRID database (version 4.4.230).

The pediatric IBD and IBD-like gene dataset revealed statistically significant enrichment of GO terms negative regulation of myeloid leukocyte differentiation (*p* = 1.85 × 10^−5^) and positive regulation of fibroblast proliferation (*p* = 4.11 × 10^−5^). The rheumatoid arthritis pharmacogenomics dataset also revealed the GO term negative regulation of myeloid leukocyte differentiation (*p* = 1.37 × 10^−5^) to be significantly enriched. The ALS mRNA dataset revealed the GO terms granulocyte chemotaxis (*p* = 1.48 × 10^−4^) and macrophage chemotaxis (*p* = 1.83 × 10^−4^) as well as several terms related to p53. Full gene ontology results are contained in Supplementary Table S2.

The extended adult complex IBD and psoriasis pharmacogenomics datasets proved too small for enrichment analysis, requiring manual evaluation of relevance to our study.

Gene ontology analysis of proteins and mRNA involved with miRNA from the NEAT1-MALAT1 block revealed only one significantly enriched GO term, mRNA cleavage and polyadenylation specificity factor complex (*p* = 8.32 × 10^−9^).

## 3. Discussion

Extending our existing dataset with ncRNA data has shown interesting new results, but also highlighted limitations and future considerations for the development of algorithms and software integrating ncRNA databases with gene ontology.

The most significant results have been obtained with the psoriasis drug response database. Based on absence in the literature and little supporting data in the lncRNA database (NPInter v5.0 http://bigdata.ibp.ac.cn/npinter5/, accessed 14 February 2024), we pruned the RP11-473M20.16 lncRNA in the resulting network. NEAT1 is through to have a role in skin inflammation processes [10] and epidermal differentiation [11], but it is also reported to be lowly regulated. In our psoriasis hypothesis, we assume NEAT1 to be down-regulated while MAP3K1-2 and AKAP13-AS1 are normally expressed. Moving from the lncRNA level to the miRNA level (see Figure 5), this leads to both hsa-miR-485-3p and hsa-miR-371a-3p lacking lncRNA-based inhibition from NEAT1 while the remaining five miRNAs are inhibited by either MAP3K1-2 or AKAP13-AS1. Finally, at the mRNA level, *TNFAIP3*, *SHOC2* and *HTR2A* are assumed to be affected by miRNA while *MAP3K14*, *ERAP1* and *SPEN* are thought to be uninhibited.

*TNFAIP3* is a known pathogenic gene in psoriasis [12] and low *TNFAIP3* expression in psoriatic skin is known to correlate with disease severity [13]. Deleterious mutations in *SHOC2* are known to cause Noonan syndrome [14], which is characterized by lentigines, a benign hyperplasia of melanocytes. *HTR2A* is more commonly investigated in association with SSRI response; however, it was also shown that *HTR2A* has a role in melanogenesis in melanoma cells and zebrafish [15]. *MAP3K14* is an NF-κB-inducing kinase and NF-κB has been long known to have a key role in inflammatory processes in psoriasis [16]. ERAP1 has been shown to regulate anti-melanocyte antibody production in psoriasis [17]. Finally, little is known about the role of *SPEN* (Msx2-interacting protein) in psoriasis. However, *SPEN* interacts with *MSX2*, which has been previously highlighted with melanoma [18,19]. In summary, the six genes highlighted in the psoriasis network are involved in skin inflammation but curiously also in melanogenesis or other melanocyte processes. Our hypothesis is consistent with clinical observations of hyperpigmentation (lentigines) following adalimumab [20] and ustekinumab [21,22] treatment. We suggest that restoration of normal skin function leads to temporary hyperpigmentation following resolution of pathogenic psoriasis skin processes.

Melanogenesis in psoriasis has been investigated in the context of pathogenesis, but no biomarkers have directly linked it to psoriasis response to biological drugs. Our findings suggest that changes in melanogenesis are rooted in miRNA-lncRNA regulatory shifts and should be further investigated in the context of pharmacogenomics and drug response. Restoration of melanogenesis or targeting processes that inhibit melanin production also appear as a potentially valid therapeutic target. Since restoration of melanogenesis in psoriasis lesions also appears to be consistent with response to drug treatment, the melanogenesis mRNA-miRNA-lncRNA network may be a promising new area for drug response biomarker discovery. We suggest further functional testing of melanogenesis in psoriasis lesions to investigate the predictive merit of melanogenesis-related mRNA, miRNA and lncRNA in predicting response to biological drugs. In particular, we suggest experimental validation of miRNAs hsa-miR-485-3p and hsa-miR-371a-3p and lncRNAs *MAP3K14*, *ERAP1* and *SPEN* in skin lesions of psoriasis patients treated with biological drugs. This may lead to the development of functional testing, which may prove more reliable than a panel of RNA biomarkers in determining responders and non-responders before the initiation of treatment with biological drugs, thus ensuring higher endpoint clinical remission rates, lower incidence of biological drug side effects and a reduced burden of health funds.

Closer investigation of genes that remain in the mRNA-miRNA-lncRNA network may reveal more interesting leads. *SLC9A3* is retained within both the pediatric monogenic and the adult complex IBD list of biomarkers. *SLC9A3* encodes solute carrier family 9 member A3 and mutations within the gene are causal for the congenital secretory sodium diarrhea 8, which may later manifest as IBD in children and young adults [23]. In complex adult IBD, *SLC9A3* is the candidate gene for the risk loci led by the SNP rs11739663 [24]. However, rs11739663 is intergenic and is not located within any known regulatory site of *SLC9A3*, thus presenting no direct causal relationship, unlike the causal variants for the pediatric IBD-like congenital secretory sodium diarrhea 8. Since impaired sodium transport has been long known to play a role in IBD pathogenesis [25], this finding might further elucidate how the intergenic variant in complex adult IBD causes pathogenesis. Rather than having a direct impact on mRNA expression, it may act via miRNA- and lncRNA-driven dysregulation of *SLC9A3* mRNA. If this is true, then NEAT1, MALAT1 as well as the miRNA block that is governed by both lncRNAs may be relevant to sodium transport impairment in adult complex IBD.

It has been shown that *KLC2*, which encodes kinesin light chain 2, is a cellular target of GSK3β and is thus capable of regulating synaptic plasticity, predominantly via trafficking of alpha-amino-3-hydroxyl-5-methyl-4-isoxazole-propionate (AMPA) receptors [26]. GSK3β inhibitors have been successfully applied in the treatment of neurodegeneration and central nerve system (CNS)-related disorders [27]. Although lithium, the first discovered GSK3β inhibitor, has shown early promise in ALS by delaying disease progression [28], later clinical trials have not been able to replicate this effect. Lithium use in ALS has insignificant effects [29] and its efficacy may be highly dependent on the genetic background of individual patients [30]. Nevertheless, GSK3β has been long known to be up-regulated in the brain and spinal cord of ALS patients [31,32] and contribute to pathogenesis, specifically by affecting neuronal metabolic integrity [33]. We speculate that in addition to aberrant action of GSK3β on *KLC2*, NEAT1 and MALAT1 lncRNAs and the miRNA block governed by them may cause dysregulation of *KLC2* in the brain and spinal cord, possibly leading to deleterious chances synaptic plasticity.

Fused in sarcoma (*FUS*) is a DNA/RNA binding protein involved in RNA metabolism and DNA repair. To this end, it is not surprising that *FUS* has a very high number of links to several lncRNAs and miRNAs within the ALS mRNA-miRNA-lncRNA network, as presented in Figure 6. *FUS* mutations are causal for highly aggressive variants of ALS named FUS-ALS [34]. We propose that in addition to missense and indel variants leading to FUS-ALS, deleterious changes in the miRNA and lncRNA landscape may lead to dysfunction of *FUS* and subsequent ALS pathology even with a healthy variant of the FUS gene.

Unfortunately, it is exceedingly difficult to construct similar hypotheses for other datasets. The main difficulty is the NEAT1/MALAT1 miRNA block, which is both crucial to network construction and also a major hurdle to clarifying results. Analysis of the NEAT1-MALAT1 block has only revealed one significant GO term. The term polyadenylation specificity factor complex is significant due to the presence of the genes *CPSF6*, *CPSF7* and *CSTF2* in the analyzed list of proteins and mRNAs that are associated with the NEAT1-MALAT1 miRNA block. *CSTF2* is cleavage stimulation factor subunit 2 while *CPSF6* and *CPSF7* are cleavage and polyadenylation specificity factor subunit 6 and 7, respectively. All three genes are involved in a complex with multiple subunits that stimulates a nonspecific polymerase with ordinarily low activity in order to elongate RNAs that bear a poly(A) signal by binding to the conventional AAUAAA hexamer and U-rich upstream sequence regions on the pre-mRNA. These three genes have been implicated in different cancer-related traits, such as breast cancer vulnerability [35], lung adenocarcinoma proliferation, apoptosis and tumorigenicity [36,37] and pancreatic ductal adenocarcinoma subtypes [38]. However, to our best knowledge, they have not yet been associated with either pediatric or adult inflammatory bowel disease, pharmacogenomic markers for biological drugs or ALS. We hypothesize that the NEAT1/MALAT1 miRNA block likely has a role in all presented datasets, but it is unlikely to be a key regulatory block in all of them. NEAT1 has been implicated in IBD intestinal inflammation by mediating TNFRSF1B [39] and exosome-mediated polarization of macrophages, thereby modulating the intestinal epithelial barrier [40]. Meanwhile, MALAT1 has been linked to the ulcerative colitis clinical subtype of IBD [41]. Similarly, both NEAT1 and MALAT1 have been suggested as clinical outcome biomarkers in rheumatoid arthritis [42]. Nevertheless, within the context of the mRNA-miRNA-lncRNA analysis presented in this article, we believe it is more likely background noise in the context of ncRNA datasets. For future research, we suggest an investigation to identify abundant and very common miRNAs and lncRNAs which often appear as background to compensate for the NEAT1/MALAT1 miRNA block and similar regulatory hotspots, such as XIST identified in the adult complex IBD loci dataset. For future algorithms, some weighting system based on expression profiles is also required to compensate for varying expression in different tissues and diseases. Alternatively, datasets need to be further curated and trimmed down to a size similar to our psoriasis pharmacogenomics dataset, either using gene ontology to filter genes covered by significantly enriched GO terms or through additional levels of evidence (ex. transcriptomic or proteomic data).

Another limitation of our study stems from its in silico design. We encourage future developments of this proposed work to include experimental data to promptly validate significant ncRNA. Future developments of the proposed workflow may also include algorithms to further narrow down the ncRNA network by checking whether an RNA biomarker is up- or down-regulated. In addition, ncRNA network analysis would benefit from analysis with high computational power and advanced algorithms that are able to take advantage of computed interaction strength or likelihood. Furthermore, the NPInter V5.0 database is to our best knowledge one of the most extensive ncRNA-ncRNA interaction databases, but it still contains a lot of high-confidence, inferred interactions that have yet to be experimentally validated.

Gene ontology results, even with extended gene lists using BioGRID, are sparce. The GO term negative regulation of myeloid leukocyte differentiation is present in both the pediatric IBD and IBD-like gene dataset and also rheumatoid arthritis pharmacogenomics. In the context of pediatric IBD, it refers to our previous findings of immunodeficiency, where the immune system is unable to initiate an appropriate response through leukocyte differentiation. In the context of rheumatoid arthritis response to biological drugs, it is instead associated with aberrant differentiation in non-responders or restoration thereof in responders to biological drugs.

Our study highlights that ncRNA databases can be used to further elucidate new processes in biomarker datasets and produce working hypotheses for targeted studies downstream. Although the emerging field faces obstacles such as appropriately dealing with miRNA and lncRNA with abundant interactions and a lack of functional annotation integration, properly developed protocols or small curated datasets are already able to overcome these shortcomings. By overcoming these challenges, we can unlock new insights into disease mechanisms, identify potential therapeutic targets, and ultimately improve patient care.

## 4. Materials and Methods

### 4.1. Interaction Databases

To date, the most comprehensive ncRNA database is NPInter V5.0 [43], which contains a wealth of experimental confirmed interactions for several kinds of ncRNAs, including lncRNA, circRNA, miRNA, snoRNA, snRNA and RNA-DNA interactions. We used the offline version of the NPInter V5.0 Experimental Validated Interactions file, accessed on 14 February 2024.

We limited our scope of search to associated miRNA and lncRNA while broadening the definition of genetic biomarkers as both mRNA and proteins. Moreover, we limited the scope of our dataset to all experimental validated interactions. Filtering was performed in R 4.1.3. using default R commands. Furthermore, we defined a filtering threshold to reduce datasets to processable sizes. If the number of occurrences for any unique biomarker is below the threshold, it is expunged (at threshold 3, unique markers that occur three times or more are retained).

In this study, we used the BioGRID database [44] to obtain gene–gene and protein–protein interactions for gene network interactor analysis in GO tools. Data were collected using the R package biogridr [45].

### 4.2. Biomarker Data

In this study, we included sets of genetic data which were already included in our previous publications. Thus, we are able to comment on the significance and relevance of the results within the frame of existing peer-reviewed works. Dataset names and summaries are listed in Table 2. Full datasets are contained in Table S1.

To test and demonstrate the new methodology on purely genetic/genomic data, we included a dataset containing causal genes for pediatric monogenic IBD and IBD-like syndromes and also the control dataset from the same publication containing adult complex IBD genetic risk loci [46].

Similarly, for pharmacogenomic data, we included two previously analyzed datasets. The first dataset contains lists of DNA, RNA and protein response biomarkers for anti-TNF therapy in rheumatoid arthritis [47]. The second dataset contains lists with DNA, RNA and protein biomarkers in psoriasis for all biological drugs indicated in psoriasis [48].

Finally, we included data from a study comparing potential overlap between known amyotrophic lateral sclerosis (ALS) genes with evolutionary-equivalent neuroregeneration genes identified in young *Monodelphis domestica*. The dataset is based on an existing curated mRNA-miRNA-lncRNA cluster [49].

### 4.3. Gene Ontology

Gene network curating and image generation and gene ontology analysis were performed using the software package CytoScape (v3.8.2., CytoScape Team) [50] with the integrated application ClueGO (v2.5.8, Laboratory of Integrative Cancer Immunology (Team 15), Paris, France) [51]. Gene sets, referred to as clusters within the ClueGO application, were constructed from genes retained after ncRNA network creation and subsequent connection number thresholding. The gene ontology analysis of mRNA-miRNA-lncRNA was defined as the results from retained genes corresponding to the mRNA in the axis. Statistical significance is set as *p* < 0.05 following integrated Bonferroni step-down correction in the ClueGO software package. ClueGO analysis was performed using the following parameters and selected options:

Ontology/pathways selected:

- Biological Process (7 February 2024);
- Cellular Component (7 February 2024);
- Molecular Function (7 February 2024).

Evidence selected: only All_Experimental.

## 5. Conclusions

Introducing gene ontology analysis to the world of ncRNA will be required in the future as disease pathogenesis models move further away from protein-based hypotheses to ncRNA-based interactive networks of dysregulation. We present a proposed workflow to take advantage of existing databases and showcase a positive example of our method with melanogenesis as predictive biological process in psoriasis pharmacogenomics or as basis for the psoriasis endotype in relation to drug response. We also highlighted the uncertain significance of NEAT1 and MALAT1 as well as the block of miRNA they are associated with. As such, we encourage further development of ncRNA-integrated gene ontology analysis of mRNA-miRNA-lncRNA networks.

## Figures and Tables

**Table 1:** NEAT1-MALAT1 block miRNA and interactors.

miRNA	hsa-mir-744-3p, hsa-miR-340-5p, hsa-miR-323a-3p, hsa-miR-208b-3p, hsa-miR-208a-3p, hsa-miR-138-2-3p, hsa-miR-1251, hsa-mir-98, hsa-miR-9-5p, hsa-mir-93-3p, hsa-miR-892c-3p, hsa-miR-877-3p, hsa-miR-7-2-3p, hsa-miR-708-3p, hsa-miR-6893-3p, hsa-miR-6884-5p, hsa-miR-6835-3p, hsa-miR-668-3p, hsa-miR-665, hsa-miR-655-3p, hsa-miR-6504-3p, hsa-miR-6088, hsa-miR-590-3p, hsa-miR-5688, hsa-miR-5590-3p, hsa-miR-543, hsa-miR-542-3p, hsa-miR-526b-3p, hsa-miR-520f, hsa-miR-520e, hsa-miR-520d-5p, hsa-miR-520c-3p, hsa-miR-520b, hsa-miR-520a-5p, hsa-miR-519d, hsa-miR-5195-3p, hsa-miR-506-3p, hsa-miR-502-5p, hsa-miR-501-3p, hsa-miR-495-3p, hsa-mir-494-3p, hsa-miR-493-5p, hsa-miR-491-3p, hsa-miR-485-3p, hsa-miR-4770, hsa-miR-4735-5p, hsa-miR-4676-3p, hsa-miR-455-3p, hsa-miR-452-3p, hsa-miR-4500, hsa-miR-449b-3p, hsa-miR-449a, hsa-miR-4458, hsa-miR-4429, hsa-miR-4319, hsa-miR-4306, hsa-miR-429, hsa-miR-4295, hsa-miR-4262, hsa-miR-425-5p, hsa-miR-424-5p, hsa-miR-423-5p, hsa-miR-422a, hsa-mir-421, hsa-miR-410-3p, hsa-miR-409-5p, hsa-miR-384, hsa-miR-382-5p, hsa-miR-381-3p, hsa-miR-379-3p, hsa-miR-378c, hsa-miR-378b, hsa-miR-378a-5p, hsa-miR-377-3p, hsa-miR-376c-3p, hsa-miR-376b-3p, hsa-miR-376a-3p, hsa-miR-374c-5p, hsa-miR-374b-3p, hsa-mir-374a-3p, hsa-miR-373-3p, hsa-miR-372, hsa-miR-371a-3p, hsa-miR-370, hsa-miR-369-3p, hsa-miR-3681-3p, hsa-mir-367-3p, hsa-miR-365a-3p, hsa-miR-363-5p, hsa-miR-362-5p, hsa-mir-34c-5p, hsa-mir-34a-3p, hsa-miR-33b-3p, hsa-miR-33a-3p, hsa-miR-338-3p, hsa-miR-335-3p, hsa-mir-331-3p, hsa-miR-329-3p, hsa-miR-328, hsa-miR-326, hsa-mir-32, hsa-mir-324-3p, hsa-miR-320d, hsa-miR-320c, hsa-miR-320b, hsa-mir-320a, hsa-miR-3139, hsa-miR-3129-5p, hsa-miR-3118, hsa-miR-30b-3p, hsa-miR-3064-3p, hsa-miR-302e, hsa-mir-302d-3p, hsa-mir-302b-3p, hsa-mir-302a-3p, hsa-miR-301b, hsa-mir-301a-3p, hsa-miR-300, hsa-miR-29a-5p, hsa-miR-299-3p, hsa-miR-296-3p, hsa-miR-23c, hsa-mir-23b-3p, hsa-miR-23a-3p, hsa-miR-224-5p, hsa-miR-22-3p, hsa-mir-223-3p, hsa-mir-222, hsa-miR-221-3p, hsa-miR-218-1-3p, hsa-miR-217, hsa-mir-216b, hsa-mir-216a-3p, hsa-miR-212-5p, hsa-mir-206, hsa-miR-205-5p, hsa-miR-202-3p, hsa-mir-200c-3p, hsa-miR-200b-5p, hsa-miR-200a-5p, hsa-mir-19a/19b, hsa-miR-199b-5p, hsa-miR-199a-5p, hsa-miR-195-3p, hsa-mir-193b-3p, hsa-mir-193a-3p/193b, hsa-miR-18b-5p, hsa-miR-18a-3p, hsa-mir-186-3p, hsa-miR-185-3p, hsa-miR-182-3p, hsa-mir-181d, hsa-mir-181c, hsa-mir-181b-3p, hsa-mir-181a/181b/18, hsa-miR-16-1-3p, hsa-miR-155-3p, hsa-miR-154-3p, hsa-mir-150, hsa-miR-149-3p, hsa-mir-148b, hsa-mir-148a, hsa-miR-146b-5p, hsa-miR-146a-3p, hsa-miR-144-3p, hsa-miR-143-3p, hsa-mir-142-3p, hsa-miR-141-3p, hsa-miR-139-3p, hsa-miR-136-3p, hsa-miR-135b-5p, hsa-miR-135a2, hsa-miR-134-5p, hsa-miR-133b, hsa-miR-132-3p, hsa-mir-130a-3p, hsa-miR-129-2-3p, hsa-miR-129-5p, hsa-mir-128, hsa-miR-125b-5p, hsa-mir-125a-3p, hsa-let-7i-5p, hsa-let-7g-5p, hsa-let-7f-5p, hsa-let-7e-3p, hsa-let-7d-3p, hsa-let-7c, hsa-let-7b-5p, hsa-let-7a-2, hsa-miR-1306-3p, hsa-miR-1271-3p, hsa-miR-1224-3p, hsa-miR-532-3p, hsa-miR-499a-3p, hsa-miR-490-3p, hsa-miR-188-3p, hsa-mir-19b-1-5p, hsa-miR-582-3p, hsa-miR-92b-3p, hsa-miR-497-3p, hsa-mir-20b-3p, hsa-miR-330-3p, hsa-mir-106b-3p, hsa-miR-145-3p, hsa-miR-15b-5p, hsa-mir-1, hsa-miR-30d-3p, hsa-mir-30c-1, hsa-miR-106a-3p, hsa-miR-96-5p, hsa-mir-92a-1-5p, hsa-mir-30a/30b/30c/, hsa-mir-20a/106b, hsa-mir-17/20b/93/51, hsa-miR-4644, hsa-miR-670, hsa-miR-613, hsa-miR-544a
Interacting lncRNA	AC009133.12, EPB41L4A-AS1, GAS5, H1FX-AS1, JPX, LENG8-AS1, LINC00324, OIP5-AS1, PCBP1-AS1, RP11-473I1.9, SLC25A5-AS1, SMAD5-AS1, SNHG1, SNHG10, SNHG11, SNHG3, SNHG5, SNHG6, SNHG7, SNHG9, XIST, SNHG16, SNHG15, DHRS4-AS1, ZFAS1, HOXB-AS1, ZNF503-AS2, MAPKAPK5-AS1, LINC00662, NDUFB2-AS1, ADPGK-AS1, SNHG17, IQCH-AS1, PROSER2-AS1, HOXA-AS2, LINC00240, LINC00910, LIMD1-AS1, TMPO-AS1, AC092159.2, LINC00511, STARD4-AS1, CIRBP-AS1, TINCR, CRNDE, SNHG11, SNORD109A
Interacting mRNA or proteins	*DDX3X*, *DNMT1*, *CDK6*, *CPSF7*, *CPSF6*, *CSTF2*, *RBM6*, *UPF1*

**Table 2:** Included dataset characteristics.

Name	Type	Biomarker Type (Source)	Number of Markers	Citation
Pediatric IBD and IBD-like genes	Genetics	DNA (PBMC)	84	[46]
Adult complex IBD loci	Genetics	DNA (PBMC)	164	[46]
Rheumatoid arthritis	Pharmacogenomics	DNA and RNA (PBMC) Proteins (PBMC, blood serum)	390	[47]
Psoriasis	Pharmacogenomics	DNA (PBMC)	64	[48]
Amyotrophic lateral sclerosis	mRNA-miRNA-lncRNA network	RNA (spinal cord tissue)	38	[49]

## Data Availability

All data are contained within Supplementary Materials or article is available upon request.

## Supplementary Materials

The following supporting information can be downloaded at: https://www.mdpi.com/article/10.3390/ijms251810210/s1.
